# Conflict management based on belief function entropy in sensor fusion

**DOI:** 10.1186/s40064-016-2205-6

**Published:** 2016-05-17

**Authors:** Kaijuan Yuan, Fuyuan Xiao, Liguo Fei, Bingyi Kang, Yong Deng

**Affiliations:** School of Computer and Information Science, Southwest University, Chongqing, 400715 China; Institute of Integrated Automation, School of Electronic and Information Engineering, Xi’an Jiaotong University, Xi’an, Shanxi 710049 China; School of Engineering, Vanderbilt University, Nashville, TN 37235 USA

**Keywords:** Wireless sensor network data fusion, Dempster–Shafer evidence theory, Belief function, Deng entropy, Evidential conflict

## Abstract

Wireless sensor network plays an important role in intelligent navigation. It incorporates a group of sensors to overcome the limitation of single detection system. Dempster–Shafer evidence theory can combine the sensor data of the wireless sensor network by data fusion, which contributes to the improvement of accuracy and reliability of the detection system. However, due to different sources of sensors, there may be conflict among the sensor data under uncertain environment. Thus, this paper proposes a new method combining Deng entropy and evidence distance to address the issue. First, Deng entropy is adopted to measure the uncertain information. Then, evidence distance is applied to measure the conflict degree. The new method can cope with conflict effectually and improve the accuracy and reliability of the detection system. An example is illustrated to show the efficiency of the new method and the result is compared with that of the existing methods.

## Background

Wireless Sensor Networks(WSN) play an important role in intelligent navigation. It can not only detect and process vehicle’s running information, such as the internal running states and external surroundings, dynamics and current position, but also transmit information via wireless, which improves the safety and the comfort of vehicles (Jiménez et al. 2012). Compared with the single detection system, WSN adopt a group of sensors to detect data (Jiménez et al. 2014). In this way, it can overcome the limitation of single sensor and enhance the accuracy and reliability of detection systems. When in face of complex environments (García et al. 2013) and other influences (Jiménez et al. 2012), the detection system of WSN can identify the object more accurately.

On account that sensor outputs may contain uncertainty, how to represent this kind of uncertain information and combine multi sensors’ outputs have attracted more and more attention. As an imprecise reasoning theory, Dempster–Shafer evidence theory (D–S evidence theory) (Dempster 1967; Shafer 1976) can be used to address the issue (Fan and Zuo 2006). D–S evidence theory was first proposed by Dempster (1967) and then developed by Shafer (1976). It is widely applied to uncertainty modelling (Walley and Cooman 2001), decision making (Fu and Yang 2014; Zavadskas et al. 2015; Mardani et al. 2015; Deng et al. 2015b; Deng 2015b), information fusion (Liu et al. 2013; Wang et al. 2015; Jiang et al. 2016) and uncertain information processing (Su et al. 2015; Yang and Han 2016). Dempster’s combination rule can allow for data fusion to combine the sensors’ reports of WSN. It takes into consideration of all sensors’ reports to make a more reasonable decision, which makes great effort to improve the accuracy and reliability of the detection system (Jiang et al. 2016).

However, different sensors’ reports may conflict highly with others due to different sources. When faced with such conflicting information, it may arrive at a counter-intuitive conclusion by using Dempster’s combination rule. How to effectually handle conflict is inevitable in data fusion of WSN (Xu et al. 2014; Moosavian et al. 2015; Yu et al. 2015). To address the issue, a number of solutions are proposed. Smets came up with an conjunctive combination rule (Smets 1990), Dubois and Prade brought up a disjunctive combination rule (Dubois and Prade 1988; Smets 1993). Murphy proposed to modify the evidences before combination (Murphy 2000), that is averaging the belief function first and combining the evidences next. Deng et al. put forward with the weighted averaging combination method to improve the Dempster’s combination rule (Yong et al. 2004). Zhang et al. (2014) introduced the vector space to deal with the issue. These solutions are generally divided into two categories, the first kind is to modify the model and the second kind is to modify the method.

This paper introduces Deng entropy (Deng 2015a) and evidence distance (Jousselme et al. 2001) and proposes a new method. First, evidence distance is adopted to quantify conflict degree among different sensors. It can be used to decrease the effect of conflicting sensors’ reports on the final decision. Besides, Deng entropy is applied to measure information volume. The more information a sensor report contains, the less possible it will conflict with others. Therefore, Deng entropy can be used to increase the effect of this kind of sensor report on the final decision. The new method takes into consideration of not only conflict degree but also information volume of sensors’ outputs. It can cope with conflict and make a reasonable decision effectually.

The paper is organized as follows. The preliminaries of D–S evidence theory and Deng entropy are briefly introduced in “Preliminaries” section. “The proposed method” section presents the new method. An example is illustrated in “Application” section to show the efficiency of the new method. Finally, this paper is concluded in “Conclusions” section.

## Preliminaries

In this section, some preliminaries are briefly introduced below.

### Dempster–Shafer evidence theory (Dempster 1967; Shafer 1976)

The Dempster–Shafer evidence theory (D–S evidence theory or belief function theory), is first proposed by Dempster (1967) and then developed by Shafer (1976). It is an imprecise reasoning theory which is widely used in the fields of uncertainty modeling (Al-Ani and Deriche 2002; Wang et al. 2015), information fusion (Molina et al. 2009; Zhang 2014; Chin and Fu 2015) and uncertain information processing (Le et al. 2007; Liu et al. 2014; Ma et al. 2016). Bayes method requires the prior information while the D–S evidence theory can deal with the uncertain information under the situation of not knowing the prior probability (Su et al. 2016, 2015). When the prior probability is known, the evidence theory can definitely degenerate to the probability theory. And it is generalized by Deng to the open world (Deng 2015c). By using Dempster’s combination rule, all the information derived from the WSN is taken into consideration and it helps to draw a more reasonable conclusion. Besides, it makes great effort to improve the accuracy of the detection system and make reasonable decisions (Yager 2004). With the requirement in optimization under uncertain environment (Du et al. 2015; Deng et al. 2015a), evidence theory is also widely used in optimization and decision making (Frikha and Moalla 2015; Han et al. 2016). Here are some basic concepts given below.

Let $\Theta$ be a set of *n* mutually exclusive and collectively exhaustive events about some problem domain, $\Theta$ is made up by all the possible answers to a question and is called the frame of discernment (Jones 2002; Yager 1987), also known as sample space, which is indicated by $\Theta =\{\theta _1,\theta _2,\ldots ,\theta _n\}$. The power set of $\Theta$ is indicated by ${2^\Theta }$, each element of which is called a hypothesis. Based on the above two concepts, the definition of belief function can be given. A belief function is a mapping m from ${2^\Theta }\,to\,\left[ {0,1}\right]$ (Jiang et al. 2015), which is defined as following:

1 $$\begin{aligned} m: 2^\Theta \rightarrow [0,1] \end{aligned}$$

satisfying

2 $$\begin{aligned} m\left( \varnothing \right) & = 0 \\ \sum \limits _{A \subseteq \Theta } m\left( A \right) & = 1 \end{aligned}$$

where *m* is also called the belief function or the Basic Probability Assignment(BPA), $m\left( A \right)$ is called the basic probability number of *A* (Dubois and Prade 1988; Jiang et al. 2015). When $m(A)>0$, A is viewed as a focal element.

The upper bound function (Dempster 1967) of a hypothesis *A* indicating the total belief degree of *A*, is denoted by *Bel*:

3 $$\begin{aligned} & Bel:{2^\Theta } \rightarrow \left[ {0,1} \right] \\ & Bel\left( A\right) = \sum \limits _{B \subseteq A} {m\left( B \right) } \quad {\forall A \subseteq \Theta } \end{aligned}$$

The plausibility function *Pl* of hypothesis *A* indicates the belief level of not denying *A*, which is defined as:

4 $$\begin{aligned} & Pl:{2^\Theta} \rightarrow \left[ {0,1} \right] \\ & Pl\left( A \right) = 1 - Bel \left( {\bar{A}} \right) = \sum \limits _{B \cap A \ne \varnothing } {m\left( B \right) }\quad {\forall A \subseteq \Theta }\end{aligned}$$

The belief function *Bel*(*A*) and the plausibility function *Pl*(*A*) represent the upper limit function and the lower limit function of hypothesis *A* respectively (Dempster 1967), satisfying $Bel\left( A \right) \le Pl\left( A \right)$.

As for the same object, there may be different evidences due to different sources of sensors, Dempster proposed to combine multi evidences by combination rule. Dempster’s combination rule, also called the orthogonal sum, is defined as following:

5 $$\begin{aligned} m\left( C \right) = {m_i}(X) \oplus {m_i}(Y) = \left\{ {\begin{array}{ll} 0 & \quad {X \cap Y = \varnothing } \\ {\frac{{\sum \nolimits _{X \cap Y = C,X,Y \subseteq \Theta } {{m_i}(X) \times {m_i}(Y)} }}{{1 - K}}}&\quad {X \cap Y \ne \varnothing} \end{array}} \right. \end{aligned}$$

*K* is the conflict factor which is adopted to measure the conflict degree. It is defined below:

6 $$\begin{aligned} K = \sum \limits _{X \cap Y = \varnothing ,\forall X,Y \subseteq \Theta } {{m_i}\left( X \right) \times {m_i}\left( Y \right) } \end{aligned}$$

Dempster’s combination rule can be used to combine two and more than two hypotheses. And when faced with more than two hypotheses, it has the following form:

7 $$\begin{aligned} m = {m_1} \oplus {m_2} \oplus \cdots \oplus {m_n} = \left( {\left( {\left( {{m_1} \oplus {m_2}} \right) \oplus \cdots } \right) \oplus {m_n}} \right) \end{aligned}$$

Though the conflict factor *K* is useful in general cases, it is not reasonable in some special cases (Zadeh 1986). Liu (2006) introduced Pignistic transformations and proposed to combine the distance between betting commitments with conflict factor *K* to measure the conflict degree.

Dempster’s combination rule is effectually in sensor data fusion in common cases. However, it may come to a counter-intuitive conclusion in some special cases. Zadeh (1986) put forward such an example that is given below.

#### *Example 1*

Assume there are three possible objects including $F_1, F_2, F_3$. The object hypotheses set is $\Theta = \{F_1,F_2,F_3\}$. Assume there are two evidences, 1 and 2, obtained by two sensors. The BPAs of objects supported by such evidences are $m_1(\{F_1\})= 0.9, m_1(\{F_3\})=0.1, m_2(\{F_2\})=0.9, m_2(\{F_3\})=0.1$. These two evidences do not support any other subsets of $2^\Theta$. The given data indicates that both two evidences have a low belief degree of 10 % supporting hypothesis $\{F_3\}$. We apply Eq. (5) directly, the BPA of hypothesis $\{F_3\}$ based on two evidences is

$$\begin{aligned} m(\{F_3\})=\frac{0.1\times 0.1}{1-0.9\times 0.1-0.1\times 0.9 -0.9\times 0.9}=1. \end{aligned}$$

The result is obviously wrong because both two evidences do not support the object $F_3$ very well.

To address the issue, Murphy (2000) proposed a different idea that averaging the belief function first and fusing the evidences next. Deng introduced the evidence distance and proposed to adopt the weighted averaging method to improve the Dempster combination rule (Yong et al. 2004; Han et al. 2007). Zhang introduced the vector space to measure the conflict degree by the distance of the space vectors (Zhang et al. 2014). This paper proposes a new method which introduces Deng entropy (Deng 2015a) to measure the information volume of the evidence. The new method considers both information volume and conflict degree, which is more reasonable in conflict management.

### Evidence distance (Jousselme et al. 2001)

The evidence distance is first proposed by Jousselme et al. (2001) and then applied to weighted averaging combination method by Yong et al. (2004). It can measure the conflict degree among evidences effectually. The concept of evidence distance is given below.

The distance between two evidence bodies ${m_1}\left( \cdot \right)$ and ${m_2}\left( \cdot \right)$ is indicated by ${d_{BOE}}\left( {{m_1}, {m_2}} \right)$, which is defined as

8 $$\begin{aligned} {d_{BOE}}\left( {{m_1},{m_2}} \right) = \sqrt{\frac{1}{2}{{\left( {{{\overrightarrow{m}}_1} - {{\overrightarrow{m}}_2}} \right) }^{\text{T}} }\underline{\underline{D}} \left( {{{\overrightarrow{m}}_1} - {{\overrightarrow{m}}_2}} \right) } \end{aligned}$$

where ${{\overrightarrow{m}}_1}$ and ${{\overrightarrow{m}}_2}$ are the vector forms of the evidence bodies ${m_1}\left( \cdot \right)$ and ${m_2}\left( \cdot \right)$ respectively (the size of each is ${2^\Theta }$). $\underline{\underline{D}}$ is a matrix of ${2^\Theta } \times {2^\Theta }$, whose elements have the following form:

9 $$\begin{aligned} \underline{\underline{D}} \left( {{s_1},{s_2}} \right) = \frac{{\left| {{s_1} \cap {s_2}} \right| }}{{\left| {{s_1} \cup {s_2}} \right| }} \quad {{s_1},{s_2} \in {2^\Theta }} \end{aligned}$$

As for multi evidences, the distances between every two evidences can be expressed in the form of distance matrix *DM*, which is given below:

10 $$\begin{aligned} DM = \left[ {\begin{array}{{llll}} 0& \quad {{d_{12}}} &\quad \cdots &\quad {{d_{1m}}} \\ {{d_{21}}} & \quad 0 & \quad \cdots &\quad {{d_{2m}}}\\ \vdots &\quad \vdots &\quad \vdots &\quad \vdots \\ {{d_{m1}}}&\quad { {d_{m2}}}&\quad \cdots &\quad 0 \end{array}} \right] \end{aligned}$$

The greater the distance of two evidences is, the less these two evidences support each other. If an evidence conflicts highly with others, it will have less effect on the final combination result. Thus, the similarity measure $Sim_{ij}$ can be defined:

11 $$\begin{aligned} Sim(m_i,m_j)=1-d(m_i,m_j) \end{aligned}$$

And the Similarity Measure Matrix (*SMM*) is expressed below:

12 $$\begin{aligned} SMM = \left[ {\begin{array}{{llll}} 1&\quad {{S_{12}}}&\quad \cdots &\quad{{S_{1m}}}\\ {{S_{21}}}&\quad1&\quad \cdots &\quad{{S_{2m}}}\\ \vdots &\quad \vdots &\quad \vdots &\quad \vdots \\ {{S_{m1}}}& \quad{{S_{m2}}}& \quad \cdots & \quad 1 \end{array}} \right] \end{aligned}$$

The support degree of each evidence is defined as following:

13 $$\begin{aligned} Sup\left( {{m_i}} \right) = \sum \limits _{j = 1,j \ne i}^m {Sim\left( {{m_i},{m_j}} \right) } \end{aligned}$$

And the credibility degree $Crd_i$ of evidence *i* is defined as following:

14 $$\begin{aligned} {Crd_i} = \frac{{Sup\left( {{m_i}} \right) }}{{\sum\nolimits _{i = 1}^k {Sup\left( {{m_i}} \right) } }} \begin{array}{{lll}} {}{} & {} {\left( {i = 1,2, \cdots ,k} \right) } \end{array} \end{aligned}$$

The credibility degree can represent how reliable an evidence is. The higher the credibility degree is, the more effect the evidence will have on the final combination result.

### Deng entropy

Deng entropy is first presented by Deng (2015a). It is an efficient tool to measure uncertain information which is the generalization of Shannon entropy (Shannon 2001; Yager 1983; Fei et al. 2015). Deng entropy can be applied in evidence theory where the uncertain information is represented by BPA. When the uncertainty is represented by probability distribution, the uncertain degree measured by Deng entropy is the same as that of Shannon entropy. The related concepts are given below.

Let ${A_i}$ be a hypothesis of the belief function $m, \left| {{A_i}} \right|$ is the cardinality of set ${A_i}$. Deng entropy $E_d$ of set ${A_i}$ is defined as following:

15 $$\begin{aligned} {E_d} = - \sum \limits _i {m\left( {{A_i}} \right) } \log \frac{{m\left( {{A_i}} \right) }}{{{2^{\left| {{A_i}} \right| }} - 1}} \end{aligned}$$

When the belief value is only assigned to single elements, Deng entropy degenerates to Shannon entropy, namely

16 $$\begin{aligned} {E_d} = - \sum \limits _i {m\left( {{A_i}} \right) } \log \frac{{m\left( {{A_i}} \right) }}{{{2^{\left| {{A_i}} \right| }} - 1}} = - \sum \limits _i {m\left( {{A_i}} \right) } \log m\left( {{A_i}} \right) \end{aligned}$$

The greater the cardinalities of hypotheses are, the greater the Deng entropy of evidence is, the more information the evidence contains. If an evidence has a great Deng entropy, it will be better supported by other evidences, and it will play a more important part in the final combination result. Here are some numeric examples to illustrate the properties of Deng entropy (Deng 2015a).

#### *Example 2*

Assume there is a mass function $m(a)=1$, the associated Shannon entropy H and Deng entropy $E_d$ are calculated as following:

$$\begin{aligned}& H = 1\times \log 1=0 \\ & E_d = -1\times \log {\frac{1}{2^1-1}}=0 \end{aligned}$$

#### *Example 3*

Given a frame of discernment $X=\{a,b,c\}$, for a mass function $m(a,b,c)=1$,

$$\begin{aligned} E_d=-1\times \log {\frac{1}{2^3-1}}=2.8074 \end{aligned}$$

For the other mass function $m(a)=m(b)=m(c)=m(a,b)=m(a,c)=m(b,c)=m(a,b,c)=\frac{1}{7}$, and the Deng entropy is calculated as following:

$$\begin{aligned} {E_d}& = - \frac{1}{7} \times \log \frac{{1/7}}{{{2^1} - 1}} - \frac{1}{7} \times \log \frac{{1/7}}{{{2^1} - 1}} - \frac{1}{7} \times \log \frac{{1/7}}{{{2^1} - 1}} \\ & \quad - \frac{1}{7} \times \log \frac{{1/7}}{{{2^2} - 1}} - \frac{1}{7} \times \log \frac{{1/7}}{{{2^2} - 1}} - \frac{1}{7} \times \log \frac{{1/7}}{{{2^2} - 1}} - \frac{1}{7} \times \log \frac{{1/7}}{{{2^3} - 1}}\\ & = 3.8877 \end{aligned}$$

Example 2 shows that Deng entropy is the same as Shannon entropy when the uncertain information is in the form of probability distribution. Example 3 illustrates that Deng entropy can measure the uncertainty effectually.

## The proposed method

In this section, a new method focusing on managing conflict and making sensor data fusion is proposed. The new method is on the basis of evidence distance and Deng entropy. The evidence distance is adopted to measure the conflict degree of sensors’ reports, and the support degree derived from the distance is used to represent the reliability of reports. If a sensor report is well supported by other reports, it will have little conflict with others, and it will be assigned to a high weight to play a more important role in the final fusion result. On the contrary, if an evidence is poorly supported by others, it will conflict highly with others, and it will be assigned to a small weight in order to have little influence in the final fusion result. The Deng entropy is applied to measure the information volume of reports (Shannon 2001). If a sensor report has a big information volume, it will be well supported by others, so that it will have a higher weight proportion. Otherwise, if an evidence has a small information volume, it may be unreliable and conflict with others. In this case, a smaller weight proportion will be assigned to it. The procedures of the proposed method are described as four steps:

- *Step 1: Calculate the support degree of evidences*

For the given data collected by sensors, using Eq. (8) to calculate the distance between every two evidences. According to Eqs. (11) and (12), the support degree *Sup*(*i*) of each evidence can be obtained.

- *Step 2: Calculate the information volume of evidences*

According to Eq. (15), the Deng entropy $E_d(i)$ of each evidence can be calculated. In Example 3, the Deng entropy of the given belief function is 0. It means the belief function has little information and may not be supported by other evidences. But it is supposed to have a little influence in the final data fusion result. To avoid that assigning zero weight to this kind of evidence, we proposed to use information volume *Iv*(*i*) to measure the uncertain information. It is defined as following:

17 $$\begin{aligned} Iv(i)=e^{E_d}=e^{- \sum \nolimits _i {m\left( {{A_i}} \right) } \log \frac{{m\left( {{A_i}} \right) }}{{{2^{\left| {{A_i}} \right| }} - 1}}} \end{aligned}$$

In this way the evidence whose total BPA is assigned to single object can have a small weight to affect the fusion result, which is more reasonable in practical application.

- *Step 3: Normalize the weights of evidences*

For each evidence, the weight $W_i$ is defined as following:

18 $$\begin{aligned} W_i=Sup(i)\times {Iv(i)} \end{aligned}$$

Assume there are *k* evidences, the normalization process is given in Eq. (19).

19 $$\begin{aligned} {w_i} = \frac{{{W_i}}}{{\sum \nolimits _{j = 1}^k {{W_j}} }} \quad {(i = 1,2, \ldots ,k)} \end{aligned}$$

- *Step 4: Make data fusion based on belief function*

Use the weights obtained by Step 3 to modify the BPAs of the evidences. Combine the weighted evidences $k-1$ times by Eq. (7) when there are *k* evidences. And then the final sensor data fusion result can be obtained.

**Fig. 1:**
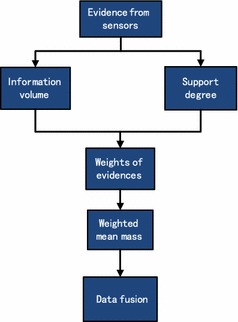
The flowchart of the new method

The specific flowchart of the new method is shown in Fig. 1.

## Application

In this section, a numerical example from reference Zhang et al. (2014) is illustrated to demonstrate the effectiveness of the proposed method. Assume that there are three objects *A*, *B*, *C* in a target recognition system. The frame of discernment is denoted by $\Theta =\{A,B,C\}$. In the WSN, there are five different kinds of sensors to observe objects which are CCD sensor $(S_1)$, sound sensor $(S_2)$, infrared sensor $(S_3)$, radar sensor $(S_4)$ and ESM sensor $(S_5)$. The evidences obtained from these five kinds of sensors are shown in Table 1.

**Table 1:** BPAs for the example

	$\left\{ {{A}} \right\}$	$\left\{ {{B}} \right\}$	$\left\{ {C} \right\}$	$\left\{ A,C \right\}$
${S_1}:{m_1}\left( \cdot \right)$	0.41	0.29	0.3	0
${S_2}:{m_2}\left( \cdot \right)$	0	0.9	0.1	0
${S_3}:{m_3}\left( \cdot \right)$	0.58	0.07	0	0.35
${S_4}:{m_4}\left( \cdot \right)$	0.55	0.1	0	0.35
${S_5}:{m_5}\left( \cdot \right)$	0.6	0.1	0	0.3

As for the BPAs given above, it is obvious that the detection of $S_2$ is abnormal. It may lead to a counter-intuitive result after fusion.

Table 2 figures out the fusion results when using different combination rules and different numbers of evidences. The calculation process about the last column of the proposed method is given below.

First, adopt Eqs. (8)–(13) to calculate the support degree *Sup*(*i*) of each evidence.

$$\begin{aligned}\hbox{Sup}(1)=3.4551\\ \hbox{Sup}(2)=2.0716\\ \hbox{Sup}(3)=3.7689 \\ \hbox{Sup}(4)=3.8239 \\ \hbox{Sup}(5)=3.8056\end{aligned}$$

Next, apply Eq. (17) to obtain the information volume *Iv*(*i*) of each evidence.

$$\begin{aligned}\hbox{IV}(1)=4.7893 \\ \hbox{IV}(2)=1.5984 \\ \hbox{IV}(3)=6.1056 \\ \hbox{IV}(4)=6.6287 \\ \hbox{IV}(5)=5.8764\end{aligned}$$

Then, obtain the weight of each evidence after normalization.

$$\begin{aligned}\hbox{w}_1=0.1827 \\ \hbox{w}_2=0.0366\\ \hbox{w}_3=0.2540 \\ \hbox{w}_4=0.2798\\ \hbox{w}_5=0.2469\end{aligned}$$

Finally, modify the BPAs by weights and combine the weighted averaging evidence 4 times. The final results are listed in Table 2.

**Table 2:** Fusion results with different combination rules

Combination rule	Fusion results			
	$\left\{ {m_1,m_2} \right\}$	$\left\{ {m_1,m_2,m_3} \right\}$	$\left\{ {m_1,m_2,m_3,m_4} \right\}$	$\left\{ {m_1,m_2,m_3,m_4,m_5} \right\}$
Dempster	m(A) = 0	m(A) = 0	m(A) = 0	m(A) = 0
	m(B) = 0.8969	m(B) = 0.6575	m(B) = 0.3321	m(B) = 0.1422
	m(C) = 0.1031	m(C) = 0.3425	m(C) = 0.6679	m(C) = 0.8578
Yager	m(A) = 0	m(A) = 0.4112	m(A) = 0.6508	m(A) = 0.7732
	m(B) = 0.2610	m(B) = 0.0679	m(B) = 0.0330	m(B) = 0.0167
	m(C) = 0.0300	m(C) = 0.0105	m(C) = 0.0037	m(C) = 0.0011
	m(AC) = 0	m(AC) = 0.2481	m(AC) = 0.1786	m(AC) = 0.0938
	$m(\Theta )=0.7090$	$m(\Theta )=0.2622$	$m(\Theta )=0.1339$	$m(\Theta )=0.1152$
Murphy	m(A) = 0.0964	m(A) = 0.4619	m(A) = 0.8362	m(A) = 0.9620
	m(B) = 0.8119	m(B) = 0.4497	m(B) = 0.1147	m(B) = 0.0210
	m(C) = 0.0917	m(C) = 0.0794	m(C) = 0.0410	m(C) = 0.0138
	m(AC) = 0	m(AC) = 0.0090	m(AC) = 0.0081	m(AC) = 0.0032
Deng et al.	m(A) = 0.0964	m(A) = 0.4674	m(A) = 0.9089	m(A) = 0.9820
	m(B) = 0.8119	m(B) = 0.4054	m(B) = 0.0444	m(B) = 0.0039
	m(C) = 0.0917	m(C) = 0.0888	m(C) = 0.0379	m(C) = 0.0107
	m(AC) = 0	m(AC) = 0.0084	m(AC) = 0.0089	m(AC) = 0.0034
Zhang et al.	m(A) = 0.0964	m(A) = 0.5681	m(A) = 0.9142	m(A) = 0.9820
	m(B) = 0.8119	m(B) = 0.3319	m(B) = 0.0395	m(B) = 0.0034
	m(C) = 0.0917	m(C) = 0.0929	m(C) = 0.0399	m(C) = 0.0115
	m(AC) = 0	m(AC) = 0.0084	m(AC) = 0.0083	m(AC) = 0.0032
Proposed method	m(A) = 0.2849	m(A) = 0.8274	m(A) = 0.9596	m(A) = 0.9886
	m(B) = 0.5306	m(B) = 0.0609	m(B) = 0.0032	m(B) = 0.0002
	m(C) = 0.1845	m(C) = 0.0986	m(C) = 0.0267	m(C) = 0.0072
	m(AC) = 0	m(AC) = 0.0131	m(AC) = 0.0106	m(AC) = 0.0039

**Fig. 2:**
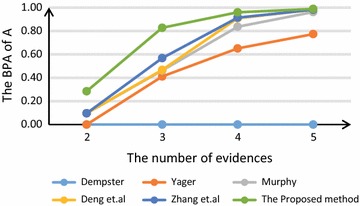
The fusion results comparison of different rules

Figure 2 compares different combination rules with different number of evidences by the BPA of object *A*. It’s clear that the proposed method has the highest belief degree of *A* whatever the number of evidences is.

Even though there are four of five evidences supporting the hypothesis $\{A\}$, Dempster’s combination rule comes to a wrong conclusion due to the conflicting evidence of $S_2$. It’s obvious that Dempster’s combination rule can’t handle with conflict.

When there are only two evidences, Yager’s method assigns most belief degree to the universal set $\Theta$ which means it can not make a decision. Other methods except for the proposed method have high belief of object *B* on account of the influence of $S_2$.

When it comes to three evidences, the first four methods can not make decisions. The reason is that the belief degree they assigned to hypothesis $\{A\}$ is smaller than 0.5. Though both Zhang’s method and the proposed method can identify the object is *A*, the belief degree to object *A* assigned by the proposed method reaches up to 0.8274 while that of Zhang’s method is only 0.5681. It’s clear that the proposed method is not only efficient but also reliable even though there are only three evidences.

Under the situation of five evidences, the proposed method improves the accuracy of identification to 0.9886. Therefore the proposed method can deal with conflict and make decision effectually.

Evidence distance reflects the relationships of different evidences. Deng entropy represents the inner properties of evidences. The proposed method takes into account of not only evidences’ relationships but also the nature of evidences so that it is efficient in dealing with conflict.

## Conclusions

In this paper, a new weighted averaging combination method on basis of evidence distance and Deng entropy is brought up to manage conflict in sensor data fusion. The proposed method has three advantages. First, it adopts Deng entropy to measure the information volume and applies evidence distance in measuring conflict degree. The new method takes into consideration of not only evidences’ relationships but also evidences’ inner properties which is more reasonable. Besides, the proposed method preserves the desirable properties of the weighted averaging method. What’s more, the new method requires less information and is much more simple to make decision compared with other methods. Generally speaking, it is an efficient method to deal with conflict in sensor data fusion and helps a lot with proper identification in WSN.
